# FIGNL1 Promotes Hepatocellular Carcinoma Formation *via* Remodeling ECM-receptor Interaction Pathway Mediated by HMMR

**DOI:** 10.2174/0115665232274223231017052707

**Published:** 2024-02-07

**Authors:** Jiabei Wang, Linmao Sun, Yao Liu, Yunguang Zhang

**Affiliations:** 1 Anhui Province Key Laboratory of Hepatopancreatobiliary Surgery, Department of Hepatobiliary Surgery, Division of Life Sciences and Medicine, The First Affiliated Hospital of USTC, University of Science and Technology of China, Hefei, 230001, China

**Keywords:** Hepatocellular carcinoma, fidgetin-like 1, hyaluronan mediated motility receptor, extracellular matrix-receptor interaction pathway, novel target, systematic treatment

## Abstract

**Background:**

The development of novel biomarkers is crucial for the treatment of HCC. In this study, we investigated a new molecular therapeutic target for HCC. Fidgetin-like 1 (FIGNL1) has been reported to play a vital role in lung adenocarcinoma. However, the potential function of FIGNL1 in HCC is still unknown.

**Objective:**

This study aims to investigate the key regulatory mechanisms of FIGNL1 in the formation of HCC.

**Methods:**

The regulatory effect of FIGNL1 on HCC was studied by lentivirus infection. *In vitro*, the effects of FIGNL1 on the proliferation, migration and apoptosis of cells were investigated by CCK8, colony formation assay, transwell and flow cytometry. Meanwhile, the regulation of FIGNL1 on HCC formation *in vivo* was studied by subcutaneous transplanted tumors. In addition, using transcriptome sequencing technology, we further explored the specific molecular mechanism of FIGNL1 regulating the formation of HCC.

**Results:**

Functionally, we demonstrated that FIGNL1 knockdown significantly inhibited HCC cell proliferation, migration and promoted cell apoptosis *in vitro*. Similarly, the knockdown of FIGNL1 meaningfully weakened hepatocarcinogenesis in nude mice. Transcriptome sequencing revealed that FIGNL1 affected the expression of genes involved in extracellular matrix-receptor (ECM-receptor) interaction pathway, such as hyaluronan mediated motility receptor (HMMR). Further validation found that overexpression of HMMR based on knockdown FIGNL1 can rescue the expression abundance of related genes involved in the ECM-receptor interaction pathway.

**Conclusion:**

Our study revealed that FIGNL1 could modulate the ECM-receptor interaction pathway through the regulation of HMMR, thus regulating the formation of HCC.

## 1 INTRODUCTION

Hepatocellular carcinoma (HCC) is a common malignant tumor in humans, and its incidence and mortality rank sixth and third in the world, respectively [1, 2]. For early-stage HCC, alternative treatment strategies are relatively mature, such as surgical resection or radiofrequency ablation [3, 4]. However, the early symptoms of liver cancer are very subtle and difficult to detect in a timely manner [5, 6]. The vast majority of patients have entered the middle and late stages once diagnosed, losing the opportunity for radical surgery [7-9]. In addition, the specific molecular mechanism of HCC is still unclear. Therefore, the study of the fundamental regulatory factors of HCC will help us better understand the regulatory mechanism of the occurrence of this disease and explore new effective therapeutic targets.

The ATPases Associated with Diverse Cellular Activity (AAA ATPase) is a crucial and essential enzyme found in almost all living organisms [10, 11]. These ATPases are part of a versatile protein family of ring-shaped P-loop NTPases, which can catalyze the hydrolysis of adenosine triphosphate (ATP) and produce inorganic phosphates [12]. Furthermore, they provide the necessary energy for processes that are crucial to various cellular physiological activities, such as protein synthesis and degradation [13, 14], DNA replication regulation [15], different signal transduction [16], and the control of gene expression patterns [16].

Fidgentin-like1 (FIGNL1) encodes a member of the AAA ATPase protein family and is related to the modulation of multiple physiological or pathological activities [17]. Firstly, FIGNL1 can be recruited to the DNA defect sites and repair DNA double-strand breaks by homologous recombination [18, 19]. Furthermore, FIGNL1 is involved in the regulation of hydrolase and ATPase activity [20, 21], microtubule depolymerization [22], reshaping of chromosome axis protein [18], bone formation [17], and meiosis [23]. Importantly, relevant studies have confirmed that FIGNL1 is associated with the development and prognosis of lung adenocarcinoma [24, 25]. However, the molecular biological role and related molecular regulatory mechanism of FIGNL1 in the occurrence and development of HCC have not been studied.

Hyaluronan-mediated motility receptor (HMMR, also known as RHAMM) is one of a relatively small number of hyaluronic acid receptors [26]. It mainly regulates the movement of cells by controlling the dynein and kinesin motor activities [27]. Many studies have confirmed the critical regulatory role of HMMR in malignant tumors [28-34]. In this study, we demonstrated for the first time the regulatory mechanism of FIGNL1 on HMMR in HCC by transcriptomic sequencing technology.

The extracellular matrix (ECM), constantly in dynamic adjustment but always in equilibrium, is mainly composed of complex structural and functional macromolecules, which play an indispensable and vital role in organ morphogenesis and the regulation of cellular physiological activities [35-39]. ECM and cell receptors directly or indirectly regulate cell activities such as differentiation [40], metabolism [41], migration [37], invasion [42], proliferation [42], and apoptosis through the mediation of transmembrane molecules such as integrins [43]. The normal physiological activities of the body require the balance between ECM and cells to be maintained, and any imbalance will lead to various pathological reactions [35, 44-49]. Therefore, one of life science's development directions is to focus on clinical protocols to maintain regular ECM-receptor interaction. Our research confirms the regulatory role of FIGNL1 on the ECM-receptor interaction pathway in HCC, providing a new promising therapeutic target for treating HCC.

As far as we know, there is no relevant research on the molecular mechanism of FIGNL1 in the formation of liver cancer. In this study, we first studied the effect of FIGNL1 on the proliferation and metastasis of HCC cells *in vitro* and validated it *in vivo*. In the meantime, this study reports for the first time the tremendous significance of FIGNL1's molecular regulatory effect on HMMR in reshaping the ECM-receptor interaction pathway, elucidating the molecular regulatory mechanism of FIGNL1 on HCC, and providing new ideas for clinical treatment of HCC.

## 2 MATERIALS AND METHODS

### 2.1 Public Data and Bioinformatics Analysis

Using the Gene Expression Profiling Interactive Analysis (GEPIA) online software (http://gepia.cancer-pku.cn/index.html), we analyzed the primary expression of FIGNL1 in a variety of different cancers. Meanwhile, GEPIA was used to analyze the FIGNL1 expression levels and the correlation between FIGNL1 expression and the overall survival time of patients. In addition, the correlation between progression-free survival and FIGNL1 expression levels in HCC patients was analyzed using the Kaplan Meier Plotter database (http://kmplot.com/analysis/index.php?p=background). The receiver operating characteristic curve (ROC) data came from the Cancer Genome Atlas (TCGA) database and was plotted with R language. The dataset GSE29721 is from the Gene Expression Omnibus database (GEO, http://www.ncbi.nlm.nih.gov/geo) and utilizing online software GEO2R (http://www.ncbi.nlm.nih.gov/geo/geo2r/) for data analysis and screening, targeting target genes with significant differences. The Cancer Cell Line Encyclopedia (CCLE, https://sites.broadinstitute.org/ccle/datasets) was used to analyze the expression of FIGNL1 in hepatocellular carcinoma cell lines. Bioinformatic analysis of transcriptome sequencing data was performed using Omicsmart, a real-time interactive online platform for data analysis (http://www.omicsmart.com).

### 2.2 Human HCC Samples

This study collected clinical samples of HCC tissues and corresponding adjacent normal liver tissues from the Department of Hepatobiliary Surgery, the First Affiliated Hospital of the University of Science and Technology of China (Anhui, China), between May 2022 and October 2022. This study has been approved by the Medical Research Ethics Committee of the First Affiliated Hospital of the University of Science and Technology of China (2022K Trial No. 291), and all patients involved in this study have provided them with written informed consent and obtained their consent. HCC cDNA microarray was obtained from Shanghai Xinchao Biotechnology Co., Ltd, containing 64 cases of HCC tissues and 26 cases of paracancer non-tumor tissues.

### 2.3 Western Blot

Cells were treated with RIPA lysate buffer (Beyotime, Shanghai, China) and cleaved on ice for one hour. Meanwhile, the BCA kit (Beyotime, Shanghai, China) was used for protein quantification. Gel electrophoresis was performed with 10% SDS-PAGE, then the protein was transferred to the PVDF membrane, sealed with 5% skimmed milk at room temperature for one hour, and incubated with primary antibody against FIGNL1 (ProteinTech Group, #17604-1-AP), β-actin (Cell Signaling Technology, #3700), HMMR (ProteinTech Group, #15820-1-AP), Caspase-3 (Cell Signaling Technology, #9662), Cleaved Caspase-3 (Cell Signaling Technology, #9661) and Bcl-2 (Cell Signaling Technology, #15071), at 4°C for 12 h. Finally, the membrane was incubated with a secondary antibody (Cell Signaling Technology, #7076 or #7074) at room temperature for one hour. The destination strips were observed using an enhanced chemiluminescence (ECL) kit (Beyotime, Shanghai, China), and the bands were analyzed using ImageJ (v1.53, National Institutes of Health).

### 2.4 Immunohistochemistry Staining

We use an immunohistochemical kit for staining. After slicing the tissue sample (0.1 mm thick), it was fixed at room temperature with 4% paraformaldehyde for 60 min. The tissue slices were placed in an oven for 2 hours at 60°C. The tissue sections were dewaxed and dehydrated with xylene and ethanol. Water washing, citrate buffer for antigen repair, and 3% hydrogen peroxide solution inactivate endogenous peroxidase activity were performed. Then, 5% goat serum (Beyotime, Shanghai, China) slices were incubated at room temperature for one hour to block non-specific binding of antibodies and then incubated overnight with FIGNL1 antibody (diluted at 1:200, #AB185674, Abcam). On the second day, the sections were incubated with HRP-labeled secondary antibody (Vector Laboratories, BA-1000) for one hour. Finally, DAB (Vector Laboratories, SK-4100) staining was performed for 3 minutes, hematoxylin restaining for 3 minutes, neutral gum was used to seal the slides, and a microscope was taken to obtain images.

### 2.5 Reverse Transcription-quantitative PCR (RT-qPCR)

RNA was extracted from the corresponding cells by the purchased RNA extraction kit (Thermo Scientific™, K0732). The RNA was reverse transcribed into cDNA according to the instructions of the purchased cDNA synthesis kit (Thermo Scientific™, A48571). The FastStart Universal SYBR Green master mix (Takara, RR820A) was used for RT-qPCR on a thermocycler (model 7300; Applied Biosystems; Thermo Fisher Scientific, Inc.). The primers of FIGNL1 and other genes are shown in Table **1**. The total reaction system for RT-qPCR is 20 ul, with each well containing 0.8 µl forward and reverse primers, 2 µl cDNA, 10 µl TB Green Premix EX Taq II, 0.4 ul 50× ROX Dye, and 6 µl nuclease-free water. At the same time, three replicates were set for each sample, and three biological replicates were set for each group of samples. The specific reaction process is as follows: 95°C for 3 min, denaturation at 95°C for 10 seconds, and annealing at 60°C for 30 seconds. The gene expression difference was calculated by the 2^-ΔΔCT^ method, and β-actin was used as the internal reference gene.

### 2.6 Cell Lines and Cell Culture

The human HCC cell lines SNU-387, HUH7, and HepG2 were purchased from Procell Life Science & Technology Co., Ltd. in March 2022. The cell line was verified in March 2022, and the follow-up experiments were performed in the same month. All cell lines we used underwent STR analysis and mycoplasma contamination testing to ensure the accuracy of the cell lines. The HEK-293T cells were from the collection of our research group. The three HCC cell lines were cultured using DMEM (Gibco™, C11995500BT) containing 10% fetal bovine serum (BI Fetal Bovine Serum, 04-001-1ACS) and 1% penicillin and streptomycin (Gibco™, 15070063). Meanwhile, the cells were cultured in a 37°C constant temperature and humidity incubator containing 5% CO_2_.

### 2.7 Lentivirus Construction and Cell Transfection

Firstly, we added the following plasmids to 293T cells for co-transfection: psPAX2, pMD2.G, and pLKO.1_shFIGNL1 containing an shRNA sequence (sh-FIGNL1#1: 5'-GCTACCATAACACCGGATCAA-3'; sh-FIGNL1#2: 5'-GCAGGATCTCAACAAACAGAT-3') that targets FIGNL1 mRNA or pLKO.1_shNT targeting control shRNA (shNT) sequence. Similarly, we constructed the FIGNL1 overexpression lentivirus and corresponding vector control lentivirus. After 48 h of co-transfection in 293T cells, the lentivirus supernatant was collected and centrifuged at 1000 RPM for 3 minutes, then it was filtered by a 0.45 mm filter. Lentivirus with FIGNL1 knockdown or overexpression and the corresponding control vector were added into the SNU-387, HUH7 and HepG2, respectively. Two days later, 10 mg/mL of puromycin (Beyotime, ST551) was added into the cell medium to screen out stable FIGNL1 knockdown or overexpression SNU-387, HUH7 and HepG2 cell lines for follow-up experiments.

### 2.8 Cell Proliferation Assay

According to the instructions, the effect of FIGNL1 on SNU-387, HUH7 and HepG2 proliferative activity was investigated using the Cell Counting Kit 8 (CCK 8) assay (Dojindo, CK04). Stable FIGNL1 knockdown and overexpression cell lines were evenly planted in a 96-well plate with a density of 3000 cells per well. On the 1^st^, 2^nd^, 3^rd^, 4^th^, 5^th^, and 6^th^ day of culture, 10 µl CCK 8 reagent was added, respectively. The absorbance of cells at 450 nm is measured using an enzyme-labeled instrument to indicate the proliferative activity of the cells.

### 2.9 Transwell Migration Assays

Cell migration assays were used to characterize the effects of FIGNL1 knockdown and overexpression on the migration ability of SNU-387 cells *in vitro*. Briefly, the treated SNU-387 cells were planted at a density of 3000 cells/well into the upper chamber of the DMEM medium without fetal bovine serum. In contrast, in the lower chamber the DMEM medium containing 10% fetal bovine serum was added. After incubating in a 37°C, 5% carbon dioxide incubator for 48 hours, the chamber was cleaned by PBS and the cells were fixed with 4% paraformaldehyde for 10 minutes. After 30 minutes of room temperature dyeing with crystal violet dye (Beyotime, C0121), a cotton swab was used to wipe out the non-migrated cells on the inner side of the chamber and then tablets were sealed with gum. Finally, the field of vision was randomly selected with a microscope and photographed to count the number of migrating cells.

### 2.10 Colony Formation Assay

Constructed stable FIGNL1 knockdown or overexpression cells were seeded into a 6-well plate at a density of 1000 cells per well. After 14 days of culture at 37°C and 5% carbon dioxide, fixed with 4% paraformaldehyde at room temperature for 15 min and then stained with crystal violet (Beyotime, C0121) at room temperature for 30 min. Images were obtained using a developer and quantitative analysis was performed using ImageJ software.

### 2.11 Transcriptome Sequencing

The whole genome transcriptome sequencing was performed on the Illumina HiSeq2500 by Gene Denovo Biotechnology Co., Ltd (Guangzhou, China). Transcriptome sequencing was used to identify mRNA transcripts with significant expression differences between FIGNL1 knockdown HUH7 cells and control HUH7 cells, as well as between FIGNL1 overexpressed HepG2 cells and control HepG2 cells. The RNA preparation and library preparation required during sequencing were carried out according to the manufacturer's instructions.

### 2.12 Reactive Oxygen Species (ROS) Analysis Using Flow Cytometry

We used the Reactive Oxygen Species assay kit (Beyotime, S0033M) to analyze the changes of Reactive Oxygen Species produced after FIGNL1 knockdown in SNU387 cells. The experimental procedure was carried out according to the kit instructions. FlowJo v10.8.1 software was used to analyze and interpret the results.

### 2.13 Cell Cycle and Apoptosis Analysis using Flow Cytometry

Cell cycle: SNU387 cells with FIGNL1 knockdown (1×10^6^ cells/well) were planted into 6-well plates. After 48 hours of culture, the cells were digested and collected with trypsin and then subjected to 70% ethanol at 4°C fixed for 2 hours. The fixed cell samples were resuspended using a mixed staining buffer of propidium iodide and RNase A (Beyotime, C1052), and incubated at room temperature in the dark for 30 minutes. Finally, samples were tested using flow cytometry. The proportion of cells in the S+G2 phase was analyzed using FlowJo v10.8.1 software.

Cell apoptosis: According to the instructions of the purchased Annexin V/PI apoptosis detection kit (Biolegend, 640922), the cells were treated in the same manner as cell cycle detection (note that cells do not need to be fixed with 70% ethanol). Similarly, flow cytometry and FlowJo v10.8.1 software were used to detect and analyze the proportion of apoptosis.

### 2.14 Animal Experiments

All animal experiments were approved by the First Affiliated Hospital of the University of Science and Technology of China (Anhui Provincial Hospital) Experimental Animal Ethics Committee (Issuing no.2023-N(A)-52). For subcutaneous xenograft experiments, ten 5-week-old male BALB/c nude mice, 5 in each group, were subjected to stable knockdown of FIGNL1 in SNU387 cells (9×10^6^ cells/mouse), and control SNU387 cells were implanted subcutaneously into the armpits of the forearms of nude mice. The length and width of the subcutaneous neoplasms were measured with a vernier caliper at 3-day intervals starting on day 7. Tumor volume was calculated according to the formula: V= 0.52× L× W^2^.

### 2.15 Statistical Analysis

We used Prism 9.0 (GraphPad) and SPSS 23.0 for graph plotting and statistical analysis, respectively. The experimental data are uniformly expressed using the mean ± standard deviation. The differences between groups were analyzed using the Student's unpaired t-test (parametric) or one-way ANOVA Sidak's post hoc test. For the paired sample data, we used the paired t-test method for statistical analysis. Comparative significance of tumor volume in mice by unpaired t-test. *P*<0.05 means a significant difference and has statistical significance.

## 3 RESULTS

### 3.1 The Expression of FIGNL1 is Up-regulated in HCC Tissues and is Associated with Poor Prognosis in HCC Patients

**Fig. (1):**
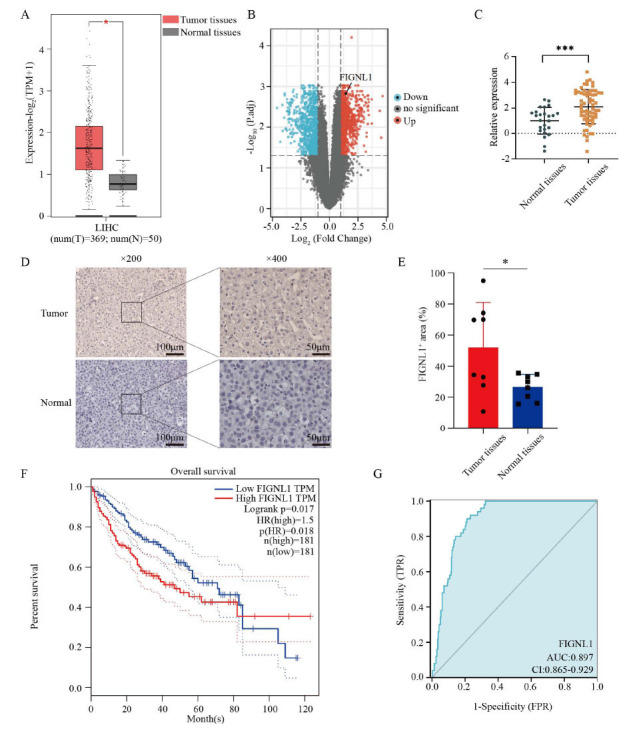
FIGNL1 is highly expressed in HCC and associated with the malignant prognosis in patients. (**A**) Boxplot illustrated the relative expression level of FIGNL1 between tumor and normal tissues in HCC based on GEPIA databases. Non-paired t-test. (**B**) The FIGNL1 relative expression level in the GSE29721 dataset. (**C**) FIGNL1 mRNA expression level analysis in HCC and normal tissues. Non-paired t-test. (**D**) The representative immunohistochemical (IHC) staining images in archival paraffin sections of patient-matched HCC *vs*. normal tissues (n = 8). (**E**) Quantification of FIGNL1 immunostaining. Paired t-test. (**F**) The correlation of FIGNL1 expression and OS in HCC patients. Log-rank test. (**G**) ROC curve analysis of FIGNL1 in HCC. Data are expressed as the mean ± SD. ns, not significant; *P< 0.05, ***P< 0.001. **Abbreviations:** FIGNL1, Fidgetin-like 1; HCC, hepatocellular carcinoma; OS, overall survival; ROC, receiver operating characteristic curve.

To screen for new potential human HCC biomarkers, we analyzed RNA expression sequencing data from the Cancer Genome Atlas (TCGA) using GEPIA and visualized the microarray data of the GEO HCC database (GSE 29721) by the GEO2R method. TCGA data revealed that FIGNL1 is upregulated in various types of human cancer (Fig. **S1A**), and compared with normal liver tissue adjacent to cancer, FIGNL1 exhibits a significantly high expression state in HCC tissue (Figs. **1A** and **B**). In order to further verify the high expression of FIGNL1 in HCC tissues, we examined the mRNA expression level of FIGNL1 using a hepatocellular carcinoma cDNA chip containing 64 cases of HCC tissues and 26 cases paracancer non-tumor tissues. The result was consistent with those shown in the above database (Fig. **1C**). Subsequently, we further confirmed the protein expression level of FIGNL1 in HCC tissues using IHC. The result indicates that the positive area of FIGNL1 is larger in hepatocellular carcinoma tissue (Figs. **1D** and **E**). To explore the potential clinical value of FIGNL1, we again used GEPIA and Kaplan-Meier Plotter databases to analyze the correlation between FIGNL1 expression levels and overall survival, progression-free survival, and clinical stage of HCC patients. As envisaged, FIGNL1 expression strongly predicts malignant outcomes in HCC patients (Figs. **1F**, **G** and **S1B**). Interestingly, we found that in the early stages of HCC development, the expression level of FIGNL1 gradually increases with the progression of HCC, although this trend disappears when the HCC progresses to the end stage (Fig. **S1C**). These results suggest that the occurrence and development of HCC may be related to the up-regulation of FIGNL1 expression, and FIGNL1 may be a new potentially valuable biomarker for human HCC.

### 3.2 FIGNL1 Facilitates the Occurrence and Development of HCC

**Fig. (2):**
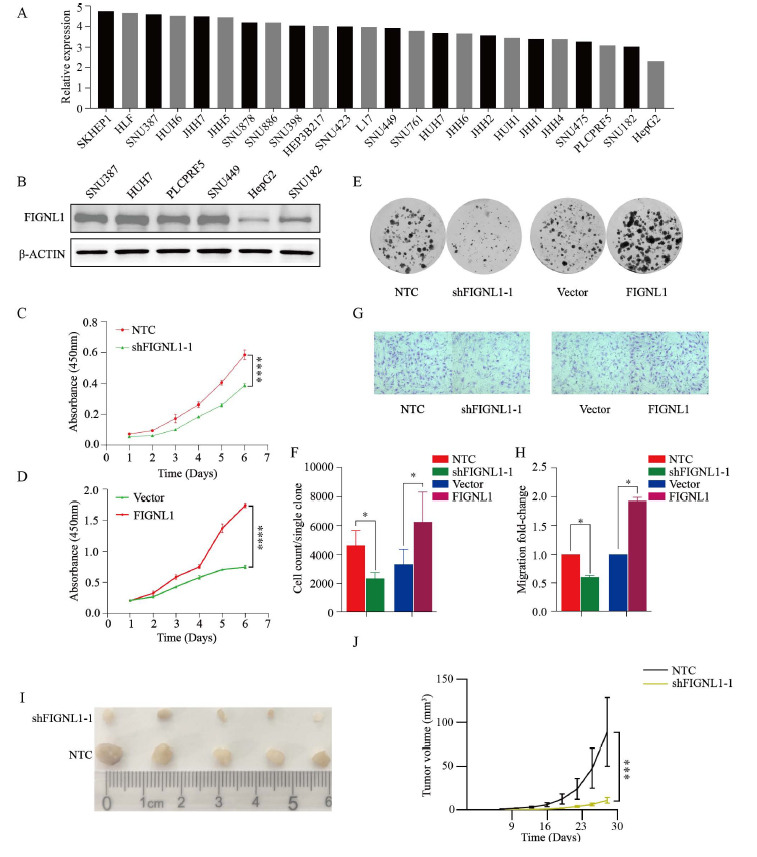
FIGNL1 can promote the occurrence of HCC both *in vivo* and *in vitro*. (**A**) Prediction of FIGNL1 expression in HCC cell lines by CCLE database. (**B**) Protein expression of FIGNL1 in the six HCC cell lines SNU387, HUH7, PLCPRF5, SNU449, HepG2 and SNU182. (**C** and **D**) CCK8 was performed to identify proliferation after FIGNL1 knockdown and overexpression in the SNU387. Student t-test. (**E** and **F**) The colony-forming ability was determined after FIGNL1 knockdown and overexpression in the SNU387. Student t-test. (**G** and **H**) Transwell assay was performed to detect the effects of FIGNL1 knockdown and overexpression on cell migration. Student t-test. (**I** and **J**) The photos of tumors and tumor growth curves were presented. Student t-test. Data are expressed as the mean ± SD. ns, not significant; *P< 0.05, ***P< 0.001 and ****P< 0.0001. **Abbreviations:** CCLE, Cancer Cell Line Encyclopedia.

In order to further explore the malignant role of FIGNL1 in the occurrence and metastasis of HCC, we first studied the effect of FIGNL1 on cell phenotype *in vitro*. Through bioinformatics prediction and WB detection, we determined that the highest expression level of FIGNL1 was found in the SNU-387 cell, followed by HUH7, and the lowest expression level was found in the HepG2 cell (Figs. **2A** and **B**). Therefore, we selected these three human liver cancer cell lines for subsequent experiments. In order to investigate the regulatory effect of FIGNL1 on human HCC cell lines *in vitro*, we have generated stable FIGNL1 knockdown (shFIGNL1) SNU-387 and HUH7 cell lines through lentiviral vector transduction expressing FIGNL1 short hairpin RNA (shRNA). Meanwhile, stable FIGNL1 overexpression SNU-387 and HepG2 cell lines were constructed by lentivirus infection (Figs. **S2A** and **B**). As previously described, RT-qPCR and WB results indicate that shFIGNL1-1 has a higher knockdown efficiency than shFIGNL1-2, so we chose this shRNA for subsequent experimental research.

CCK8 and colony formation experiments showed that knockdown of FIGNL1 in SNU-387 cell significantly inhibited its proliferative ability *in vitro* compared to the control group, while overexpression of FIGNL1 could promote the proliferative activity of SNU-387 cell (Figs. **2C**-**F**). Moreover, transwell experiments showed that the knockdown of FIGNL1 significantly downregulated the migration ability of SNU387 cells (Figs. **2G** and **H**). Similarly, the upregulation of FIGNL1 increased the migration ability of SNU-387 cells *in vitro*. The above results suggest that FIGNL1 is related to the malignant phenotype of SNU387 cells and has a significant regulatory effect on the activity of human liver cancer cells *in vitro*.

In order to explore the regulatory effect of FIGNL1 on HCC *in vivo*, we further confirmed the promotion effect of FIGNL1 on HCC *in vivo* by constructing subcutaneous xenografts in nude mice. In the xenotransplantation experiment, the tumor growth rate and volume of the shFIGNL1 group were smaller than those of the control group (Figs. **2I** and **J**). This conclusion strongly supports the malignant significance of FIGNL1 in the occurrence and development of HCC *in vivo*.

### 3.3 Knockdown of FIGNL1 has Blocking and Promoting Effects on Cell Cycle and Apoptosis, Respectively

**Fig. (3):**
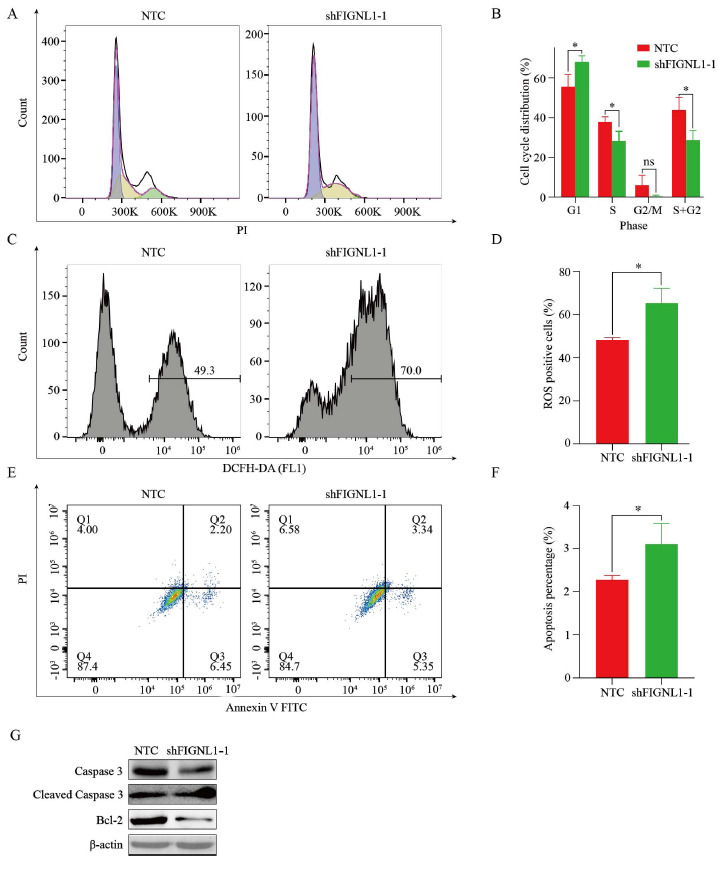
FIGNL1 is associated with cell cycle progression and apoptosis. (**A** and **B**) Cell-cycle distribution was measured by PI staining in HUH7 cells transfected with shFIGNL1-1 and the control, followed by flow cytometric analysis. Student t test. (**C** and **D**) ROS was detected using DCFH-DA by flow cytometry in HUH7 cells transfected with shFIGNL1-1 and the control. Student t test. (**E** and **F**) Cell apoptosis was measured by FITC-Annexin V and PI staining in HUH7 cells transfected with shFIGNL1-1 and the control, followed by flow cytometric analysis. Student t test. (**G**) The protein expression of Caspase-3, cleaved Caspase-3 and Bcl-2 were analyzed in HUH7 cells transfected with shFIGNL1-1 and the control. Data are expressed as the mean ± SD. ns, not significant; *P< 0.05. **Abbreviations:** PI, propidium iodide.

To further investigate the regulatory effect of FIGNL1 on the growth of human liver cancer cells, we used flow cytometry to further explore the effects of FIGNL1 on cell proliferation and apoptosis. Compared to the NTC group, FIGNL1 knockdown caused a greater proportion of cells to be blocked in the G0/G1 phase of the cell cycle process. In contrast, the proportion of cells in the S+G2 phase is significantly reduced (Figs. **3A** and **B**), indicating that the ability of cell division and proliferation was significantly weakened. In order to verify whether the knockdown of FIGNL1 attenuates the proliferation capacity of human liver cancer cell lines by inducing apoptosis, we first detected the ROS activity of FIGNL1 knockdown cells by flow cytometry (Figs. **3C** and **D**). Further, we measured the apoptosis ratio using an Annexin V-FITC/PI apoptosis double staining kit. The results prove that FIGNL1 knockdown significantly promoted the apoptosis rate of SNU-387 cells compared with the control group (Figs. **3E** and **F**). In addition, the results of apoptosis-related proteins (Caspase-3, Cleaved Caspase-3, and Bcl-2) further revealed the mechanism of shFIGNL1-induced apoptosis of human hepatoma cells (Fig. **3G**). Compared with the control group, the expression of Cleaved Caspase-3 in the shFIGNL1-1 group was upregulated, while the expression of pro Caspase-3 was opposite, and the expression of anti-apoptotic protein Bcl-2 decreased. The above results suggest that FIGNL1 is likely to influence the occurrence and development of HCC by utilizing its regulatory role in cell proliferation and apoptosis.

### 3.4 FIGNL1′s Positive Regulatory Effect on HMMR Plays an Essential Role in the Formation of HCC

**Fig. (4):**
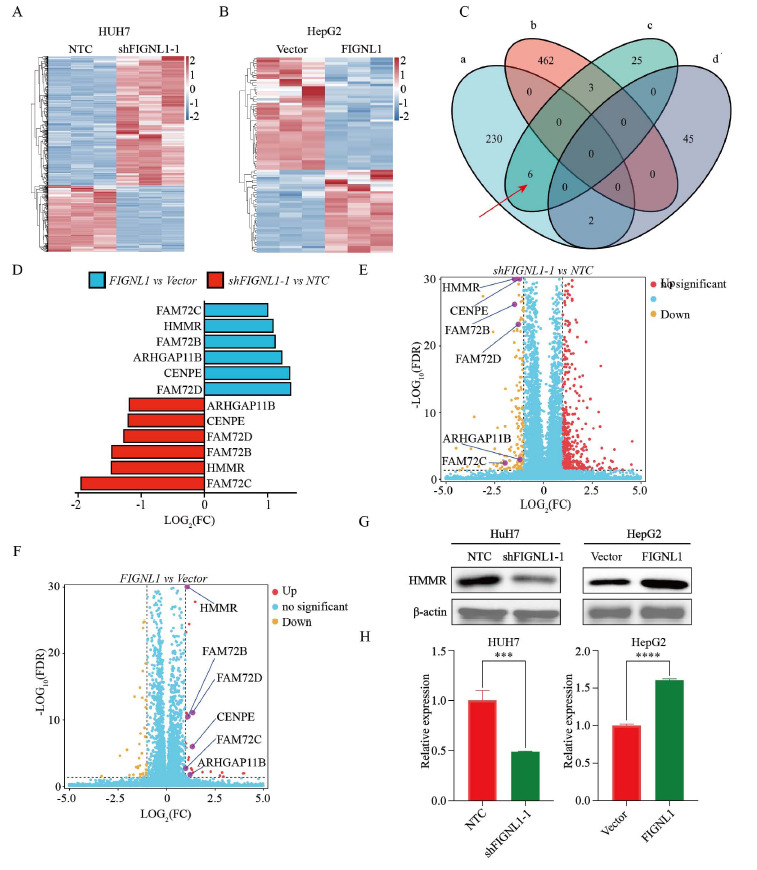
FIGNL1 has a positive regulatory effect on HMMR. (**A**) Cluster heat map analysis of significantly different genes of FIGNL1 knockdown in HUH7. (**B**) Cluster heat map analysis of significantly different genes of FIGNL1 overexpression in HepG2. (**C** and **D**) Six genes were identified through the intersection of down-regulated genes of shFIGNL1 *vs* NTC(a), up-regulated genes of shFIGNL1 *vs* NTC(b), up-regulated genes of FIGNL1 *vs* Vector(c) and down-regulated genes of FIGNL1 *vs* Vector(d). (**E**) Volcano of altered genes in shFIGNL1 *vs* NTC from transcriptome sequencing in HUH7. (**F**) Volcano of altered genes in FIGNL1 *vs* Vector from transcriptome sequencing in HepG2. (**G** and **H**) The protein and mRNA expression of HMMR were analyzed in HUH7 cells transfected with shFIGNL1-1 and the control. Student t test. Data are expressed as the mean ± SD. ns, not significant; ***P< 0.001 and ****P< 0.0001. **Abbreviations:** HMMR, hyaluronan mediated motility receptor.

To further investigate whether the molecular mechanism of FIGNL1 promotes the occurrence and development of HCC, we conducted transcriptomic sequencing using stable HUH7 cells that knocked down FIGNL1 and HepG2 cells that overexpressed FIGNL1, and their corresponding control group cells. The results showed that there were significant differences in 703 genes between the shFIGNL1-1 group and the control group, including 465 up-regulated genes and 238 down-regulated genes (Figs. **4A** and **E**). Compared with the vector group, 81 genes in the FIGNL1 overexpression group were significantly different, including 34 up-regulated genes and 47 down-regulated genes (Figs. **4B** and **F**). We did not screen any differential genes in the intersection of the up-regulated genes in the shFIGNL1-1 group and the downregulated genes in the overexpression group. In the same way, by crossing the downregulated genes in the shFIGNL1-1 group with the up-regulated genes in the overexpression group, we found that there were six genes with significant differences, namely HMMR, CENPE, ARHGAP 11B, FAM72B, FAM72C, and FAM72D (Figs. **4C** and **D**). Gene ontology analysis found that these six DEGs were mainly enriched in Binding and Cellular Anatomical Entities. Furthermore, these two Go terms rank most prominently in Molecular Function (MF) and Cellular Com-ponent (CC), respectively (Figs. **S3A** and **B**). Based on known research work, we found that only HMMR is involved in the occurrence and development of tumors. After analyzing HCC data from the TCGA database using the GEPIA online website, it was found that HMMR also showed a significantly high expression state in HCC (Fig. **S3C**), which showed a significant positive correlation with the expression of FIGNL1 (Fig. **S3D**). In order to further verify the regulatory effect of FIGNL1 on HMMR, WB and RT-qPCR experiments were conducted, and the results were consistent with the above experimental conclusions (Figs. **4G** and **H**). The above data support our belief that the positive regulatory effect of FIGNL1 on HMMR plays a critical potential role in the formation of hepatocellular carcinoma.

### 3.5 FIGNL1 Facilitates the Progression of HCC by Remodeling the ECM-receptor Interaction Pathway through HMMR

**Fig. (5):**
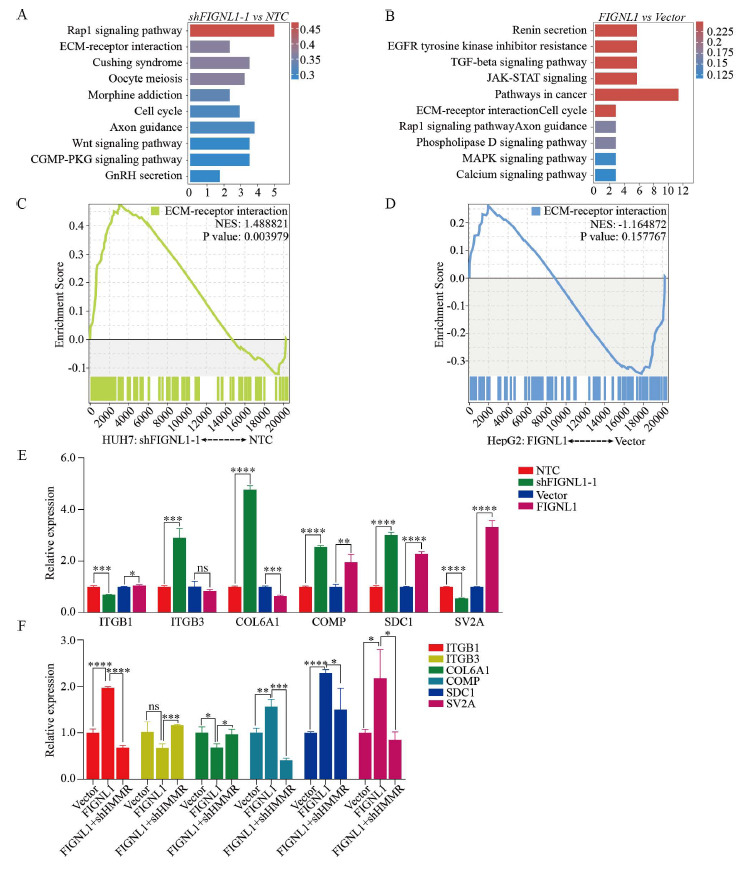
HMMR mediates the remodeling effect of FIGNL1 on the ECM-receptor interaction pathway. (**A**) KEGG pathway enrichment analysis of altered genes in shFIGNL1 *vs* NTC from transcriptome sequencing in HUH7. (**B**) KEGG pathway enrichment analysis of altered genes in FIGNL1 *vs* Vector from transcriptome sequencing in HepG2. (**C**) Single-gene GSEA pathway analysis of HMMR in shFIGNL1 *vs* NTC from transcriptome sequencing in HUH7. (**D**) Single-gene GSEA pathway analysis of HMMR in FIGNL1 *vs* Vector from transcriptome sequencing in HepG2. (**E**) The mRNA expression of ITGB1, ITGB3, COL6A1, COMP, SDC1 and SV2A were analyzed in the FIGNL1 knockdown and overexpression group, respectively. Student t test. (**F**) The mRNA expression of ITGB1, ITGB3, COL6A1, COMP, SDC1 and SV2A were analyzed in the Vector, FIGNL1 and FIGNL1+ shHMMR group in HepG2. Student t test. Data are expressed as the mean ± SD. ns, not significant; *P< 0.05, **P< 0.01, ***P< 0.001 and ****P< 0.0001.

Further research on transcriptomic sequencing data showed that in the KEGG pathway enrichment analysis of significantly different genes, whether the FIGNL1 knockdown group or the overexpression group, the ECM-receptor interaction pathway showed significant enrichment (Figs. **5A** and **B**). In addition, single-gene GSEA pathway analysis of HMMR, CENPE, ARHGAP11B, FAM72B, FAM72C and FAM72D revealed that only HMMR could be enriched, while the remaining five genes were not enriched in any pathway (Figs. **5C** and **D**). Coincidentally, in GSEA pathway analysis, the pathway enriched by HMMR is precisely the ECM-receptor interaction pathway. Through in-depth analysis of the ECM-receptor interaction pathway, we found that HMMR is an important regulator involved in this pathway and plays an essential physiological role in maintaining the normal functioning of this pathway. In addition, in order to further verify the regulatory effect of FIGNL1 on the ECM-receptor interaction pathway, we used RT-qPCR technology to analyze the changes of the other six genes involved in this pathway, including ITGB1, ITGB3, COL6A1, COMP, SDC1 and SV2A. The results indicated that the knockdown and overexpression of FIGNL1 can alter the ECM-receptor interaction pathway (Fig. **5E**). To investigate whether HMMR mediates the influence of FIGNL1 on ECM-receptor interaction pathway remodeling, we used HepG2 human liver cancer cell line to knock down HMMR on the basis of FIGNL1 overexpression. Subsequently, we tested the six genes (ITGB1, ITGB3, COL6A1, COMP, SDC1 and SV2A) involved in the ECM-receptor interaction pathway and found that the knockdown of HMMR significantly rescued the impact of FIGNL1 overexpression on the ECM-receptor interaction pathway (Fig. **5F**). These results demonstrate that FIGNL1 can reshape the ECM-receptor interaction pathway through the mediation of HMMR, thereby playing a critical molecular regulatory role in the occurrence and metastasis of HCC.

## 4 DISCUSSION

At present, hepatocellular carcinoma (HCC) is still one of the malignant tumor diseases that perplex humanity, and it still maintains a high incidence rate and mortality situation all over the world [50, 51]. Despite the availability of surgery [52], radiotherapy [53], chemotherapy [54], immunotherapy [55], and other treatment options, the 5-year survival rate of patients with hepatocellular carcinoma, especially those with advanced hepatocellular carcinoma, is still very low [56-58]. Exploring new therapeutic targets may help us step up a new level in the treatment of hepatocellular carcinoma [59-61].

ATPases Associated with Diverse Cellular Activity (AAA ATPase) are related to various cellular activities [10, 14, 62-66]. They are necessary enzymes in all living organisms and play an essential role in different physiological processes such as DNA replication and repair [67], protein synthesis and degradation [14], microtubule formation and depolymerization [68], signal transduction [69], and other pathological activities. As a member of this protein family, FIGNL1 is involved in the above physiological processes [18, 19, 22, 23, 70, 71]. Recently, relevant studies have reported the role of FIGNL1 in the tumorigenesis of lung adenocarcinoma (LUAD) and other tumors [24, 25, 72, 73], but its function in HCC malignancy has yet to be said.

This study aims to study the molecular role and regulatory mechanism of FIGNL1 in the regulation of HCC occurrence and development. Here, for the first time, we found that FIGNL1 is highly expressed in human HCC and has a significant or partial correlation with the malignant prognosis and tumor stage of patients, indicating that FIGNL1 has the ability to become a strong predictor of the progression and prognosis in HCC patients. In addition, the knockdown of FIGNL1 by shRNA *in vitro* can significantly weaken the proliferation, migration, and invasion abilities of SNU387 cells, affect cell division, increase the proportion of apoptosis, and block the number of cells in the G0/G1 phase of the cell cycle. Upregulation of the expression of FIGNL1 has the opposite effect. At the same time, experiments further confirmed the promotion effect of FIGNL1 on the occurrence and development of HCC *in vivo*. In summary, FIGNL1 has excellent potential to become a new and promising potential biomarker for the treatment and prognosis of HCC.

We use Transcriptome sequencing technology to deeply explore the specific molecular regulatory mechanisms of FIGNL1 in promoting the malignant progression of HCC. The results showed that there was a significant correlation between FIGNL1 and various genes related to the interaction of ECM receptors. In the intersection of down-regulated and up-regulated genes in the FIGNL1 knockdown group and the overexpression group, only the ECM receptor interaction pathway involving HMMR was found in the KEGG pathway enrichment analysis. Consistent with this result, we found significant enrichment of the ECM-receptor interaction pathway in KEGG pathway enrichment analysis in both the FIGNL1 knockdown and overexpression groups. Further research on some genes (ITGB1, ITGB3, COL6A1, COMP, SDC1 and SV2A) involved in the ECM receptor interaction pathway further confirmed that FIGNL1 regulates the remodeling of the ECM-receptor interaction pathway through HMMR.

It is well known that extracellular matrix components around tumor cells play an essential regulatory role in solid tumors' formation, progression, and treatment resistance [74-76]. Sufficient evidence supports that extracellular matrix components can effectively change the proliferation, metastasis, and invasion ability of tumors and have a far-reaching impact on the prognostic effect of various therapeutic approaches [74, 77]. Our study shows that FIGNL1 has a significant remodeling effect on the extracellular matrix, which is worthy of our attention. Immunotherapy has a broad prospect in the treatment of malignant tumors, and extracellular matrix microenvironment homeostasis is regarded as a highly dynamic partner of the immune system. Its rich protein components and immunoactive molecules play an indispensable role in immune regulation in both the homeostasis and pathological states of the body. Therefore, further study of FIGNL1 may also help to further integrate the extracellular matrix with tumor immunotherapy. Now, the regulation function of FIGNL1 on the remodeling of ECM receptor interaction pathway through HMMR further supports the indispensable role of FIGNL1 in the occurrence, progression, and treatment of HCC, laying the foundation for its use as a new target for HCC treatment. The development of related inhibitors targeting the new treatment target FIGNL1 may be a unique and effective treatment strategy for HCC patients.

## CONCLUSION

This study demonstrated for the first time that the FIGNL1-HMMR-ECM receptor signaling axis regulates the formation of HCC, which provides a basis for future research and development of molecular-targeted drugs targeting this signaling axis.

## AUTHOR'S CONTRIBUTIONS

YL and YZ designed the study. JW and LS performed the experiments. JW wrote the manuscript. YZ analyzed and verified the integrity of the data. All authors contributed to the article and approved the submitted version. All authors approved the manuscript and agreed to be accountable for all aspects of the research.

## Figures and Tables

**Table 1:** Primer sequences used for gene expression analysis.

**Gene**	**Forward (5'**-**3')**	**Reverse (5'**-**3')**
FIGNL1	CTAGGAGCTAGTAGATCCCGAG	GATGTGCTGGTTCTGTAGGTCC
HMMR	GGCTGGGAAAAATGCAGAGGATG	CCTTTAGTGCTGACTTGGTCTGC
ITGB1	GGATTCTCCAGAAGGTGGTTTCG	TGCCACCAAGTTTCCCATCTCC
ITGB3	CATGGATTCCAGCAATGTCCTCC	TTGAGGCAGGTGGCATTGAAGG
COL6A1	GCCTTCCTGAAGAATGTCACCG	TCCAGCAGGATGGTGATGTCAG
COMP	GGAGATGCTTGTGACAGCGATC	TGAGTCCTCCTGGGCACTGTTA
SDC1	TCCTGGACAGGAAAGAGGTGCT	TGTTTCGGCTCCTCCAAGGAGT
SV2A	CGCCTTTCCTTCTGTGTTTGCC	GAGAACACTCGCTCAGGATGTC
β-actin	CACCATTGGCAATGAGCGGTTC	AGGTCTTTGCGGATGTCCACGT

**Abbreviations:** FIGNL1, Fidgetin Like 1; HMMR, Hyaluronan Mediated Motility Receptor; ITGB1, Integrin Subunit Beta 1; ITGB3, Integrin Subunit Beta 3; COL6A1, Collagen Type VI Alpha 1 Chain; COMP, Cartilage Oligomeric Matrix Protein; SDC1, Syndecan 1; SV2A, Synaptic Vesicle Glycoprotein 2A.

## Data Availability

The datasets used and/or analyzed during the current study are available from the corresponding author upon reasonable request.

## LIST OF ABBREVIATIONS

CCLE: Cancer Cell Line Encyclopedia
COL6A1: Collagen Type VI Alpha 1 Chain
COMP: Cartilage Oligomeric Matrix Protein
ECM: Extracellular Matrix
FIGNL1: Fidgetin-like 1
GEPIA: Gene Expression Profiling Interactive Analysis
HCC: Hepatocellular Carcinoma
HMMR: Hyaluronan Mediated Motility Receptor
ITGB1: Integrin Subunit Beta 1
ITGB3: Integrin Subunit Beta 3
OS: Overall Survival
PI: Propidium Iodide
ROC: Receiver Operating Characteristic Curve
SDC1: Syndecan 1
SV2A: Synaptic Vesicle Glycoprotein 2A
TCGA: Cancer Genome Atlas
