# Supplementary material for: FIGNL1 Promotes Hepatocellular Carcinoma Formation via Remodeling ECM-receptor Interaction Pathway Mediated by HMMR
Source: Curr Gene Ther. 2024 Feb 7;24(3):249–63. doi: 10.2174/0115665232274223231017052707 (PMC11071652; doi:10.2174/0115665232274223231017052707)
Supplement: Supplementary file 1 [file CGT-24-249_SD1.pdf]

Supplementary Material

FIGNL1 Promotes Hepatocellular Carcinoma Formation *via* Remodeling ECM-receptor Interaction Pathway Mediated by HMMR

Jiabei Wang[1], Linmao Sun[1], Yao Liu[1,*] and Yunguang Zhang[1,*]

[1]*Department of Hepatobiliary Surgery, The First Affiliated Hospital of USTC, Division of Life Sciences and Medicine, University of Science and Technology of China, Anhui Province Key Laboratory of Hepatopancreatobiliary Surgery, Hefei, 230001, China*

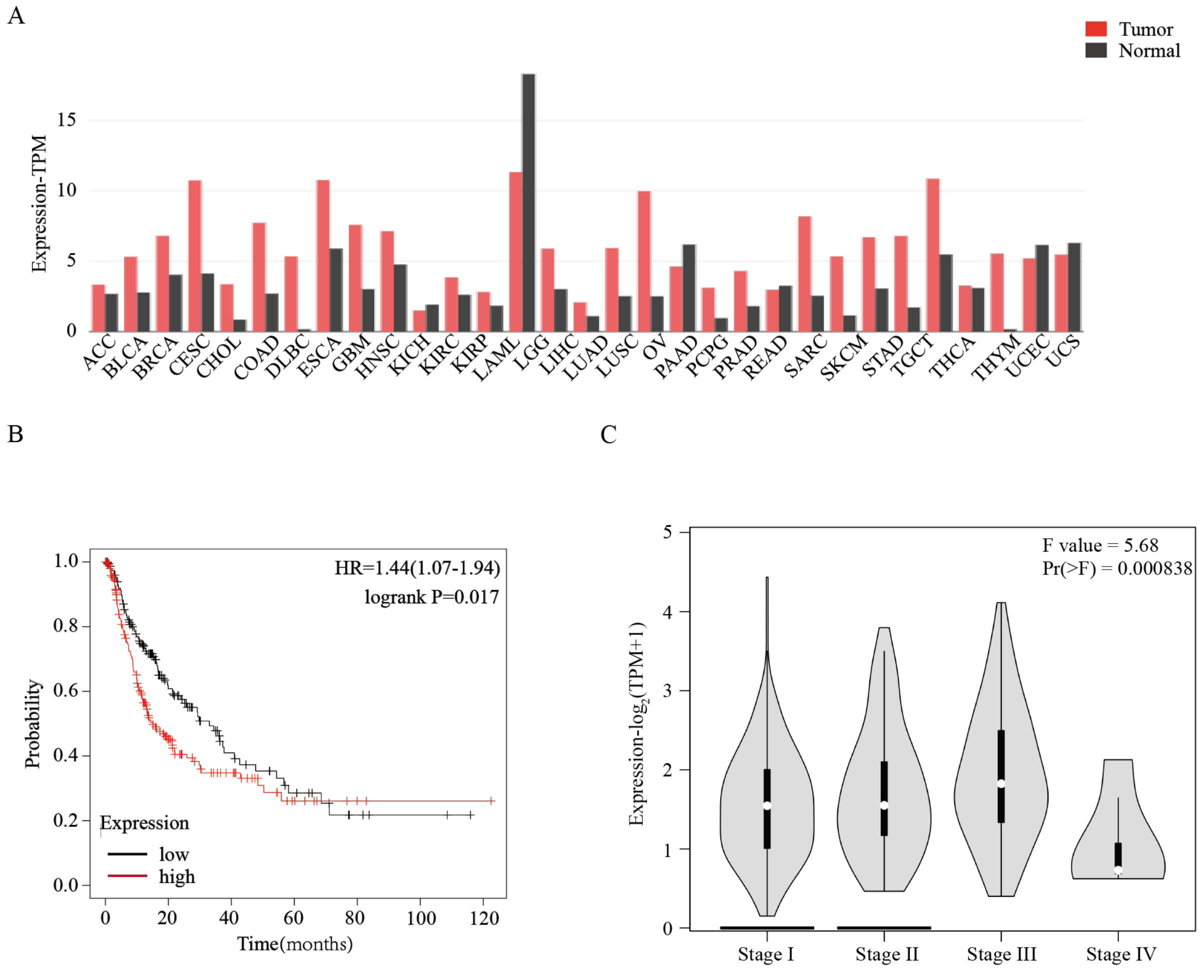

Supplementary Fig. (1). Expression analysis of FIGNL1 in public database. (**A**) The expression of FIGNL1 in various types of cancer. (**B**) Correlation analysis between the expression of FIGNL1 and PFS in HCC patients. Log-rank test. (**C**) In the early stage of HCC, the expression of FIGNL1 gradually increases as HCC progresses. Data are expressed as the mean ± SD. ns, not significant; $^{*}P < 0.05$, $^{**}P < 0.01$, $^{***}P < 0.001$ and $^{****}P < 0.0001$. PFS, progression free survival. (*A higher resolution / colour version of this figure is available in the electronic copy of the article*).

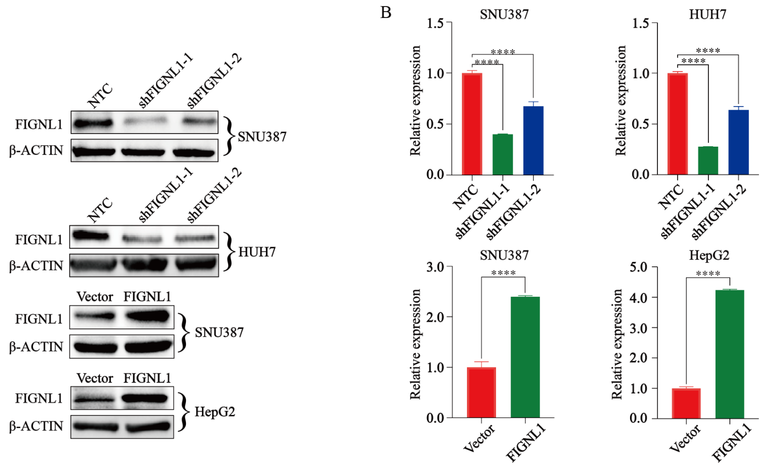

Supplementary Fig. (2). Construction of FIGNL1 knockdown and overexpression human liver cancer cell lines. (**A**) The protein and (**B**) mRNA expression analysis of the efficiency of FIGNL1 knockdown and overexpression in SNU387, HUH7 and HepG2 cells. Student t-test. Data are expressed as the mean ± SD. ns, not significant; *P< 0.05, **P< 0.01, ***P< 0.001 and ****P< 0.0001. (*A higher resolution / colour version of this figure is available in the electronic copy of the article*).

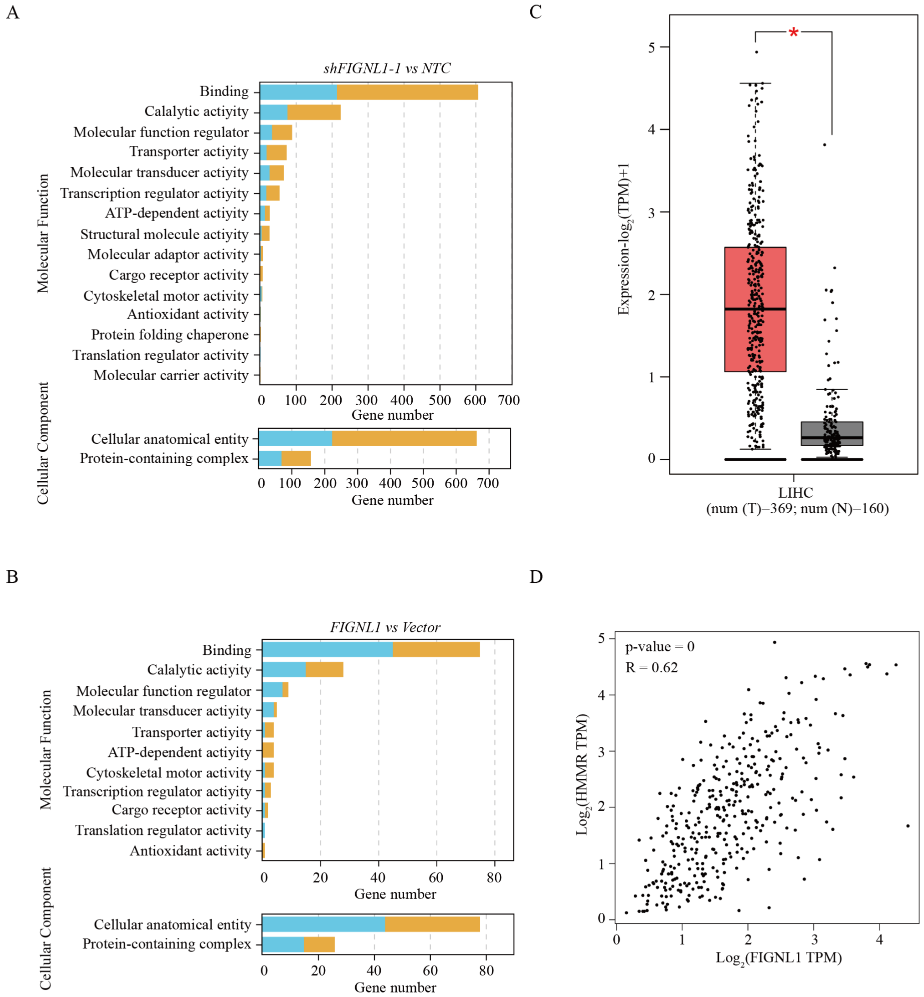

Supplementary Fig. (3). Gene ontology analysis of altered genes from transcriptome sequencing and the expression analysis of HMMR in HCC. (**A**) Gene ontology analysis of altered genes in shFIGNL1 *vs* NTC from transcriptome sequencing in HUH7. (**B**) Gene ontology analysis of altered genes in FIGNL1 *vs* Vector from transcriptome sequencing in HepG2. (**C**) Boxplot illustrated the relative expression level of HMMR between tumor and normal tissues in HCC based on GEPIA databases. Non-paired t-test. (**D**) The correlation of FIGNL1 expression and HMMR in HCC patients. Pearson Correlation Analysis. Data are expressed as the mean ± SD. ns, not significant; *P< 0.05, **P< 0.01, ***P< 0.001 and ****P< 0.0001. (*A higher resolution / colour version of this figure is available in the electronic copy of the article*).
